# Three Years with the Army of the Potomac—A Personal Military History

**Published:** 1911-09

**Authors:** William Warren Potter

**Affiliations:** Buffalo, N. Y., Brevet Lieutenant Colonel, U. S. Volunteers ; Surgeon in Charge of First Division Field Hospital Second Army Corps; Surgeon 57th Regiment New York Volunteers; Assistant Surgeon 49th Regiment New York Volunteers ; Recorder Second Division Hospital Sixth Army Corps, etc., etc.


### Three Years with the Army of the Potomac—A Personal Military History

By William Warren Potter, M.D., Buffalo, N. Y., Brevet Lieutenant Colonel, U. S. Volunteers; Surgeon in Charge of First Division Field Hospital Second Army Corps; Surgeon 57th Regiment New York Volunteers; Assistant Surgeon 49th Regiment New York Volunteers; Recorder Second Division Hospital Sixth Army Corps, etc., etc.

Routine camp life now went on for several days, during which time we heard of Grant's victory at Shiloh. Lieutenant Hickmott preferred charges against me about this time for alleged neglect of duty, in refusing to attend one of his men in the night, who shot himself purposely, as I believed, to escape duty, causing only a slight flesh wound of no special importance. The weather was rainy and unpleasant up to April 11, when the sun once more appeared; but, meanwhile, the roads had become almost impassable, rendering it extremely difficult to get up our stores, which were brought from Newport News, eighteen miles distant, in wagons, twenty-four hours being required to accomplish the distance.

Fatigue parties in large numbers were sent out to corduroy the roads, and a highway was finally constructed to Ship Point on the James, seven miles distant, where a depot of supplies was established. On the 11th I took two loads of sick back to Young's Mills, where a hospital had been established in the rebel barracks left there, and Dr. Russell, of the 5th Vermont, placed in charge. Our position is now before Lee's Mill opposite the enemy's right, which is protected by water, dammed at intervals to deepen the stream. On the 16th a reconnoissance was made at Dam No. 1, between Lee's and Wynn's Mills, where General Smith tried to force a passage and attack the works, or develop their strength. He did the latter. Brook's Brigade of Vermont troops was assigned to the duty, our brigade in support. It proved unsuccessful as far as getting a foothold in the works, but some important information was obtained. The 3d Vermont lost seventy-five men, of whom twenty-two were killed. I saw General McClellan on the field in conference with General Smith about four o'clock P. M. We returned to our camp about six o'clock P. M. without casualty.

Colonel Bidwell returned to the regiment on the 18th, looking thin and pale, having lost about fifty pounds of flesh during his illness. On the 19th the 49th took its turn in support of the batteries for the night, and I went along to look after any sick or wounded who might require medical services. Lieutenant William McLean of the 5th U. S. cavalry, formerly of Lancaster, accompanied me, and we busied ourselves trying to keep dry, for it was a rainy night, whiling away the time as best we could in story telling and smoking. McLean's squadron was assigned to duty with General Smith, and, as there was very little opportunity to use cavalry in our present position, he became a frequent visitor in our camp, many of the officers of the 49th being his acquaintances. He was a genial companion, a good campaigner, and a valuable officer. Lieutenant, afterwards General, Custer often came with him and spent an hour or two with me. They were fast friends, and both brave even to audacity. The night referred to, when we were out with the battery supports, was a most inclement one, dark and, at intervals, the rain fell in torrents. The pickets became alarmed several times during the night, and blazed away at each other, the batteries occasionally belching forth their deep bass intonations, to vary the musical (?) sounds. The flashes of the guns athwart the deep darkness of the night, simulated lightning, and the bullets flew over our heads at times in great numbers, sounding like hailstones against the great forest trees. It was a most uncomfortable night, yet we got back to camp next morning without casualty. I was, however, nearly sick for two or three days afterwards, though I very soon recovered my wonted vigor. I had not yet been off duty a day by reason of illness since entering the service, this being the nearest thereto that I had yet come.

On April 23, Governor Sprague, of Rhode Island, paid General Davidson a visit, accompanied by General Stoneman of General McClellan's staff, and a troop of cavalry. He wore a military cap, blouse, and sword-belt, and attracted considerable attention by reason of his prominence as a "War Governor," who had equipped and sent out a regiment at his own expense, when the government experienced its first trial. After partaking of General Davidson's hospitality for nearly an hour, the party proceeded on its tour of inspection. About this time several of the subaltern officers resigned and there was considerable disaffection in the regiment; but it disappeared very soon after the siege was raised.

Large siege guns were now being brought up and placed in position, and operations were pushing with vigor. Dr. Hall returned on the 28th, much to my gratification and relief, for, since we left Camp Griffin, I had been in medical charge of the regiment, and felt the burden of my responsibility to be rather heavy, particularly during so much activity of movement. I received visits during these days from Dr. Frank H. Hamilton, Dr. Joseph Robinson, of Hornellsville, and Lieutenant Charles O. Shepard, of the 82d N. Y. On the 24th an order was received designating the camp as "Winfield Scott"—the name to apply throughout the army during the siege. While we were here my court-martial trial, on the charges preferred by Lieutenant Hickmott, was called at Brigade Headquarters one day; I went up, with Quarter-Master Tillinghast as counsel, to defend the case, but, for some reason or another, the complainant failed to appear, and the Judge Advocate dismissed the case. The paymaster appeared on the last of the month and paid us for January and February; but I discounted my rolls for March and April, in addition, and thus obtained four months' pay, the first I had received since the November and December payment. This enabled me to discharge all my indebtedness and send $250.00 to my wife.

May 1. We are comfortable for the first time since leaving Camp Griffin, as Dr. Hall brought two tents with him from Washington, which we have appropriated to our own comfort, using one in part for the official headquarters of the surgeons. He brought four other tents also, which have been distributed amongst the field and staff officers, so we are all very well provided with quarters. I took my trousers off tonight on going to bed, the first time I have done so in six weeks. We are but half a mile from the rebel lines, no fires are allowed after dark, and not a drum has been sounded for a month. If our precise position were known to the enemy, we should probably be shelled out, or, to say the least, be kept in a constant tension of dread of such possibilities. From three to five men are shot daily within a thousand yards of our camp, but the 49th has been fortunate thus far in not losing a man, since our losses of the first day we came up here. Firing is constantly heard on all sides, chiefly at working parties of our inclining or theirs. Saturday night, May 3. The batteries all along the lines are firing every two or three minutes, mostly from siege mortars and 100 pound Parrott's. Everything points to a general bombardment soon, but the firing tonight appears to be simply practice for range. Seven mortars are within a short distance of our camp, two of which were placed in position today. We turn out in line of battle from one to three times a day, and nearly every morning at daylight there is an alarm of some kind, which brings the whole division to arms. Lieutenant McLean stayed all night with us again last night, a thing which he is in the habit of doing frequently, and we enjoy his visits very much. He generally brings some important news whenever he comes, which is always acceptable.

WILLIAMSBURG.

When we awoke Sunday morning, May 4, we found the enemy had evacuated his whole line of works during the previous night, and appeared to be in full retreat upon Richmond. We were put in immediate pursuit, and passed through their fortifications which were very formidable. In their deserted camps were found quantities of rations and camp equipage, including flour in barrels, beans, camp kettles, a few tents, and the like. Baggage wagons in large numbers were also secured, and many stragglers were made prisoners. We passed General Magruder's headquarters about ten o'clock A. M., and overtook the skirmishers of their rear guard at two P. M., or thereabouts. Our division had the advance, and the 49th was the second or third regiment in column. About noon I was sent back to our late camp with some orders, and, returning, caught up with the troops about three o'clock P. M. By dark we had driven the skirmishers well into Williamsburg, the 6th cavalry suffering a loss of probably fifty killed and wounded. I dressed one of the wounded by the wayside in the twilight. We rested on our arms that night in line of battle in a wheatfield; Colonel Alberger and I seeking the shelter of a little clump of trees nearby, and spreading our blankets together for the night. We were awakened about four o'clock A. M. by rain falling in our faces, and at daylight the troops were up and in line, though it rained as hard as ever it could pour.

Hooker, whose division had come up in the night and was placed on our left, pushed his skirmishers forward into the woods beyond at daylight, and soon found the enemy strongly entrenched behind a slashing of heavy timber. The fighting began at once, and kept up all day. At ten o'clock Hancock's Brigade with two regiments of ours, by a detour to the right, was swung around on the left flank of the enemy, capturing two deserted redoubts which commanded the position in front of Hooker. As soon as this was discovered Magruder ordered six regiments to charge Hancock's line, which they did in right gallant style. Hancock reserved his fire until they were well upon him, then delivered it fairly in their faces, driving them back in great confusion with heavy loss. It seems that Hancock had asked to have the remainder of the division sent to his support, but Sumner, who was in command, refused to comply. Meanwhile, word was sent to General McClellan, who had remained at Yorktown to superintend the shipment of Franklin's Division by water to West Point, of the situation early in the afternoon, and at four o'clock I saw him ride upon the field, accompanied by the Prince de Joinville, the Count of Paris, the Duc de Chartres and others of his staff, the whole party literally covered with mud. Smith was promptly at his side and obtained consent to join Hancock; we were off at once, but by the time we got there the affair was over, and darkness was upon us as well. I saw the body of Lieutenant Colonel Baddam, of the 5th N. C., lying in one of the redoubts, his forehead pierced by a bullet. He was a young man, handsome, and of splendid physique. He was heard to say, just at the climax of the charge, "Now we've got the d—d Yankees, give them Bull Run and Ball's Bluff." I stood around a fire all night, for it rained hard, and tried to dry my clothing and keep warm, but made no attempt to lie down and sleep. The men slept in the rain and mud as best they could, and when morning came we found the works abandoned and the enemy in full retreat.

The field was covered with the enemy's dead, and we had over one hundred of his wounded in our front to care for. I went over the field Tuesday morning, the 6th, and helped gather up the wounded which we carried to a large barn nearby, where the same afternoon I assisted in making the operations. Here we amputated ten legs and several arms, the most serious cases having been left on our hands by the hastily retreating foe. I visited Williamsburg on the 7th, where I saw between four and five hundred more of their wounded, distributed to the various churches and William and Mary College. Twelve surgeons were sent into our lines by Longstreet, including his medical director, Dr. Cullen, to look after these wounded. I had an opportunity to talk with some of the prisoners, which was the first time that experience had come to me, and also obtained some of their currency which I sent home. They say very little, though enough for me to infer that the men—the private soldiers—don't take as great heart in the war as their officers.

McClellan paid our command a visit, and complimented Hancock and his troops, on their soldierly conduct and success. In going over the field again on the 8th I saw dead rebels lying all through the woods behind logs and stumps, and graves were thick and numerous. This was my first experience in going over a battle-field immediately after the action, and it impressed me greatly. Our own dead were very well buried, but the enemy's were more hastily and imperfectly done. The wounded of Smith's division, together with those of the enemy which fell into our hands, were placed on board the steamer "Commodore" and sent to Washington. The wounded in Williamsburg—four or five hundred—were left there in care of the rebel surgeons, who were sent into our lines for that purpose. Some of the doctors were quite intelligent looking men, but most of them were shabbily dressed, and were only to be distinguished from civilians by the letters "M. S."—signifying medical staff—on their hats. Williamsburg itself is a sleepy old village of probably two thousand inhabitants in time of peace, many of whom, however, had now retired with the rebel army, so it was quite deserted except by the army of temporary occupation. A State Lunatic Asylum is located here which, together with William and Mary College, affords it some distinction. Lieutenant McLean paid us another visit on the 8th, and was in his usual good spirits.

### THE MARCH UP THE PENINSULA.

Friday, May 9, we started at six o'clock A. M. and marched nineteen miles over the road on which the rebels retreated, camping for the night at, or near, Burnt Ordinary, a small country P. O. We passed broken wagons stuck in the mud, some of them partly burnt, caissons, and other war material which was dropped in the haste of retreat, and our cavalry brought back squads of prisoners captured from the rear guard. At Burnt Ordinary I "captured" a large quarto volume of Quain's anatomical plates, from the house of a doctor which the cavalry had gone through. The book I found in the yard, and still have it in my possession as a part of my library. (Now in the library of the University of Buffalo.) Saturday, May 10, we marched fifteen miles, starting before five o'clock in the morning, and at night were said to be but thirty-two miles from Richmond. Smith's division had the advance, our brigade moving in front. We camped for the night on a field where the cavalry had an engagement the day before, and our pioneers buried eighty dead —sixteen of our own men, and sixty-four of the enemy. The men are constantly capturing stray horses, and Dr. Hall and I have taken one in partnership to carry our extra blankets, forage, rations, and the like.

On the 11th, Sunday, it was rumored that Adjutant Bullymore, who left us on the 3d, while we were before Yorktown, had died at Fort Monroe. This rumor was, unhappily, confirmed by a letter from Quartermaster Tillinghast the same evening. This was a sad blow to us all, for Bullymore was a promising young officer, whose friends were legion. Our double sorrow consisted in losing our genial Quartermaster, H. D. Tillinghast, who went to Fort Monroe to take Bullymore, and also to have his teeth repaired. He went to Buffalo with Bullymore's remains, sickened and, on May 29th, died there; so he never came back to us. The loss of these two officers from the staff, so suddenly and so near together, shocked us in the extreme, and was a damaging blow to the regiment as well.

We are now near New Kent, C. H., and on the night of the 11th, I went to West Point, twelve miles distant, with a load of sick for shipment to Yorktown. I was out all night, returning to camp just as reveille was sounding. Harbeck, our genial sutler, resigned about this time and I sent my porcupine fish by him, when he went home. William Woodruff was appointed sutler in Harbeck's place, and, though a good business man, he was not as popular as his predecessor. On the 14th I was attacked with neuralgia in the face, and was off duty until the 16th from that cause. We were them at the White House, where we remained until the 19th. The Custis Estate, where we were encamped, was formerly owned by General R. E. Lee; it was here that George Washington courted and married his wife, and it is one of the finest plantations I have yet seen. While we were here the 5th and 6th provisional corps were formed, commanded respectively by Porter and Franklin. Smith's becomes the 2d division of the 6th corps, Slocum's division being the 1st.

On the 19th we marched nine miles farther towards Richmond, being now but sixteen miles in direct line from that city. We passed through some beautiful country today, untouched hitherto by the devastation of war,—fields of grain nearly ripe, fine residences, and elegant plantations. Tonight for supper we had duck, eggs, and strawberries, all the products of the country. We moved five miles on the 20th, and still live on the country principally—I speak of our own mess—sweet potatoes (new), eggs, and chickens being the chief articles of our cuisine at present. We engaged another, and I hope a better cook today—one who formerly cooked for General Smith, and who understands his business thoroughly.

Thus far into the bowels of the land have we marched without serious impediment, and are under orders to march again at three o'clock A. M. tomorrow, our brigade to take the lead. Our mails are very irregular now, and it is three days since we had the last one; so we are pretty hungry for news from home. A march of eight miles on the 21st brought us to Cold Harbor, a spot destined to become historic from the fact that two of the severest battles of the war were to be fought in its vicinity. It is simply a crossroads, with a country inn, and a smithy. General Stoneman sent up a balloon about two miles in advance of us this morning, to reconnoitre the enemy's position. Major Johnson is not well these days, but I hope he is not seriously threatened. He is a brave man and a sterling officer—it would be a pity to lose his valuable services (he afterwards became Lieutenant Colonel of the 49th, and was mortally wounded in the defense of Washington, July 12, 1864, dying a few days afterwards). I am in good health and spirits myself, and the regiment is reputed one of the healthiest in the division, and the strongest in the brigade. Dr. S. S. Mulford, of the 33d, visits me often, and he is in good health and spirits also. He is a good campaigner, and an excellent companion.

### MECHANICSVILLE.

We remained at Cold Harbor until Friday, May 23, when the 3d brigade made a reconnoissance towards Mechanicsville, supporting General Stoneman's cavalry. Late in the afternoon, after marching about five miles, a battery opened upon us. Our artillery soon silenced it after exchanging a few shots, when we crossed a creek and slept on our arms that night, without anything to eat. Next morning, the 25th, we stood to arms at daybreak, soon advanced and drove the enemy out of Mechanicsville in a drenching rain, the brigade losing three men killed, and five or six wounded, mostly from the 33d and 77th regiments. Our regiment did not lose a man, though it was under fire for an hour or more. Mechanicsville is a hamlet five miles north of Richmond, consisting of half a dozen houses, most of which suffered more or less from our artillery fire. We took possession of a beautiful grove in the rear of the principal residence, and there remained over Sunday. Our supplies reached us at night and we made ourselves quite comfortable again, though we were without tents. During Sunday, the 25th, we picketed one side of the Chickahominy, and the enemy the other. The river runs in a valley, the hills rising on either side. At intervals during the day we saw carriages containing ladies, on the opposite hills, who had come out from Richmond to get a sight of the hated Yankee troops. I am in possession of a saddle captured from a "Secesh" cavalryman at Mechanicsville, which comes in play for our extra horse. On Monday, May 26, bright and early we were relieved by another brigade, and returned to a position near Gaines's Mill, four miles east of Mechanicsville. So the first Mechanicsville affair was at an end. I visited the place in the autumn of 1886, and sat down upon a log in the same grove in which we passed Sunday, May 25, 1862. The Century Magazine for June, 1885, on page 313 gives a good illustration of our conquest of the place. The artillery referred to in the title of the picture is the artillery which the 3d brigade of Smith's division was supporting, as I have described.

The end of May was now fast approaching, and it finally came without special incident to the 49th. It rained hard on the afternoon and night of the 26th, and on the 27th the entire regiment was sent out on fatigue duty, chopping trees for timber to build a road across the low ground on either side of the river, apparently preparatory to crossing. The pontoons went down to the river bank on the night of the 26th, and a bridge will doubtless be laid very soon. I picked up a snake skin near my tent this morning, which I propose to send home as a relic, and as indicating the two-fold character of the danger we are in. Heavy firing was heard all the afternoon of the 27th, in the direction of Mechanicsville, but we are unadvised as to its cause.

Dr. Hall's health is quite poor, and has been so for some time, which adds to my duties and responsibilities very much. Major Johnson is, however, in better health than he was a few days ago, and I trust will soon be as strong as ever. Dr. S. B. Hunt wrote a letter from Fort Munroe, which was published in the Buffalo papers, in which he speaks of having seen Tillinghast at Old Point; the papers have just been received, and we have been much interested in reading the doctor's newsy account of his visit with "Till." I see Dr. Hamilton quite often these days, as he is medical director on Franklin's staff, and so comes around both socially and officially. On the 28th we had news of Porter's fight at Hanover Court House, which accounts for the firing we heard yesterday. Dr. Herrick sent a pail of lemons for the sick today, which was a comfort as the boys are fagged. May 29. I visited General Smith's headquarters today, where I saw a lot of prisoners captured by Porter on the 27th. Major Chapin, of the 44th N. Y., was wounded in that fight. (He subsequently became Colonel of the 116th, and was killed at Port Hudson in 1863. Chapin Place, out near the Park, is named in honor of him.)

May 31. We have not moved since we came to this camp (near Gaines's Mill) last Monday morning, which is the longest time we have spent in any one place since we left Yorktown. Today we hear firing over the Chickahominy on the left of the army, which sounds like a heavy engagement, and at night the shells burst over the tree tops, so we can see them quite plainly. Tom Dewey arrived in camp today, having been commissioned 2d Lieutenant in Company A under Captain Cluney. He is a valuable acquisition to the line officer list, and will, no doubt, distinguish himself. I went down to Dr. Gaines's today to see the rebel wounded whom Porter captured at Hanover C. H.; they are a sorry lot, and two rebel surgeons were caring for them as best they could.. A severe thunder storm prevailed all last night, and one artilleryman is reported killed by lightning at Mechanicsville.

Sunday, June 1. The battle which began yesterday on our left, was renewed this morning and lasted until noon. It is known on our side as the Battle of Fair Oaks—the enemy call it Seven Pines. Surely, the Oak and Pine were contending. We have just received the news of the evacuation of Corinth, and all were very much surprised. The inquiry on every hand is, "Where have the devils gone?"

I saw Professor Lowe, the balloonist, yesterday and had quite a long conversation with him. His balloon is stationed near us. He tells me that he saw the rebel troops the day before, moving towards the field of Fair Oaks, and so apprised the commanding general. Major Washington, of Johnson's staff, was captured by our pickets a day or two since, and some important battle plans were found upon his person. This Sunday afternoon I went down below Dr. Gaines's, where there is an open field looking across the river, towards the scene of yesterday's battle, and on which Ayers's and Griffin's batteries are posted in battery. Both Ayers and Griffin were lying on the ground, directing the artillery practice and chatting about the war, and particularly of the rebel commanders. "Smith G. W." was the familiar name they gave General Gustavus W. Smith, who had succeeded to the command at Fair Oaks, upon the wounding of General Joseph E. Johnson. All day yesterday I was occupied with my monthly report, which must always be sent in promptly, lest there be complaints from headquarters. Tuesday, June 3. We were under orders to be ready to move at a moment's notice—not an unusual order nowadays. The whole regiment was startled today, by the receipt of the sad intelligence of the death of Quartermaster H. D. Tillinghast, at Buffalo, whither he had gone with Adjutant Bullymore's remains. Nothing could have surprised us more than this sad news; for it is but a month today since he left us well, excepting some trouble with his teeth which he went to Fort Monroe to have looked after. Mr. T. had the respect of both officers and men, and was not only a popular and efficient officer, but socially he was the best of companions, and he bore the name of "Gentleman" stamped upon his every act.

The rebel General Pettigrew, who was wounded on Saturday, has been brought to this side of the river, and is at Dr. Gaines's house where I am contemplating a call upon him. I sent home today a copy of the "Illustrated News," containing pictures of the hospital—barns at Williamsburg. I procured the admission of the artist inside the barns while we were operating, and was administering chloroform in one of the sketches which he made of the operations.

(Continued.)

